# Why Being Physically Active or Inactive Affects Older Women's Physical Role?

**DOI:** 10.1155/2021/6687381

**Published:** 2021-02-24

**Authors:** Pedro Jesús Ruiz-Montero, Laura Rubio, Cristina G. Dumitrache, Óscar Chiva-Bartoll

**Affiliations:** ^1^Department of Physical Education and Sport, Faculty of Education and Social Sciences, Campus of Melilla, University of Granada, Melilla 52006, Spain; ^2^Department of Developmental and Educational Psychology, University of Granada, Granada 18011, Spain; ^3^Department of Education and Specific Didactics, Faculty of Humanities and Social Sciences, Universitat Jaume I, Castellón 12071, Spain

## Abstract

**Background:**

Active aging is aimed at promoting quality of life in older adults. Nevertheless, the relationship between physical role and the practice of physical activity (PA) can be influenced by bodily pain feeling and by a low level of health-related quality of life (HRQoL). Passive and active strategies are susceptible to being modified and constitute an important psychological predictor of adaptation to pain. This cross-sectional study (1) analyzed the differences between inactive/active older adult women in terms of clinical and sociodemographic characteristics, pain coping strategies, and HRQoL; (2) studied the associations between pain coping strategies, the dimensions of the HRQoL questionnaire, and physical role; and (3) determined if passive strategies, bodily pain, physical function, and general health were significant mediators in the link between being inactive/active and physical role.

**Methods:**

Participants of the present cross-sectional study completed measures of clinical and sociodemographic characteristics, HRQoL using the Short-Form Health Survey-36, and active and passive strategies using the Vanderbilt Pain Management Inventory (VPMI).

**Results:**

A total of 157 inactive (69.9 ± 7.1 years) and 183 active (68.8 ± 5.3 years) women from rural areas were included in the study. Both groups significantly differed in the majority of the clinical and sociodemographic characteristics measured, pain coping strategies, and HRQoL. Bodily pain, physical function, and general health predicted physical role. Moreover, passive strategies, bodily pain, physical function, and general health mediated the link between inactive/active participants and physical role.

**Conclusions:**

Being physically active or inactive contributes to a better understanding of the link between PA, pain coping strategies, and physical role in older women.

## 1. Introduction

The increase in the number of older adults has been one of the achievements of the last decades and has promoted a growing interest in finding out factors related with improving wellbeing in older people. Active aging has been defined as “the process of optimizing opportunities for health, participation, and security in order to enhance quality of life as people age” [1]. Therefore, staying active during aging can be related with healthy and successful aging.

Sedentary behavior has become an emerging public health concern among population age 54 years old and older. In developed countries, a majority of the population does not reach the minimal recommended levels of physical activity (PA) [2]. This, in turn, promotes negative health consequences that could hinder daily life activities performance and a decrease of health-related quality of life (HRQoL) [3, 4]. Physical inactivity is especially worrying in the case of women, since they have 50% less likelihood to take part in physical training than men [5]. Therefore, it is crucial to analyze the different variables that are related with PA and its potential benefits for older women [6].

When HRQoL is assessed using the Short-Form Health Survey-36 (SF36), physical role, defined as the extent to which physical health interferes with work and with other daily activities, is a dimension related to limitations in performing daily activities as a result of physical health problems that includes lower performance that the desired one, limitation in the type of activities carried out or difficulty in carrying out activities [7]. As it seems that, among the different ways to improve older women's physical role, exercise can be a fundamental strategy to achieve this, further research is needed to understand the characteristics of the association between physical role and PA [8].

The association between an active lifestyle and physical role in older adult women could be mediated by many variables; among them, physical function stands out as an important one. Physical function is defined as the capacity of performing PA such as self-care, walking, climbing stairs, bending down, catching, or carrying weights [7]. Physical functional abilities impair with age, and many studies have investigated these changes and their consequences for work performance associated to physical role [9–11]. Since physical role relies enormously on physical function capacities, it is essential to have a good physical function as we get older, so that a better physical role can be achieved. The maintenance of overall functioning is essential for the aging individuals in order to remain independent, to prevent the development of various diseases and chronic illnesses, to reduce the risk of fall-induced injuries, and to increase overall mood and satisfaction with life [8].

Many studies recognize that PA is key to preserving physical function for performing daily life activities [12–15]. Moreover, structured PA enhances HRQoL, perceived physical, social, and emotional functioning [16]. In this sense, it is reasonable to believe that through physical exercise, physical function and physical role will improve [17].

Another variable that could possibly influence the relationship between the likelihood of practicing physical exercise and physical role is bodily pain. The impact of bodily pain on older adults' daily lives increases during the aging process, and it is associated with the risk of experiencing chronic pain [18], as well as the decrease of some HRQoL dimensions such as physical role.

On the other hand, consequences of chronic pain are significant; thus, people who suffer them try to cope with them by using passive strategies such as social support seeking. An example of this passive strategy is when chronic pain patients turn to other people, such as their physician, in a hope they can help them control their pain experience. However, sometimes, patients who suffer from chronic pain are tired of managing their feelings, and they tend to catastrophize the situation by interpreting the situation as being terrible [19, 20]. It is possible that people who experience pain on a daily basis tend to anticipate that they might experience pain if they perform moderate-vigorous PA [21], and in turn, they become more sedentary. Nevertheless, it has been found that physical activity or being active influences general coping strategies and pain coping strategies used to cope with pain [22–25].

Pain catastrophizing, a passive strategy, is a pain-related cognition susceptible to being modified and an important psychological predictor of adaptation to pain [26, 27]. Catastrophizing during old age is related with depressive symptoms, which in turn affects older adults' HRQoL, functional impairment, and chronic illness [28, 29]. For instance, it has been found that the use of passive strategies negatively affects physical role [30]. In addition, physical role, which is related to better mobility, a high rate of PA, and daily life activities performance, contributes to diminishing catastrophizing [31].

As we have shown, PA helps maintaining a better psychological status and contributes to effective pain coping, and better general health [32] and physical function [33]. Moreover, the relationship between being physical active and general health is associated with a better physical condition of participants from PA interventions [6, 33]. On the other hand, inactive older people normally show a worse general health than those who are active in their daily life [34].

**Figure 1:**
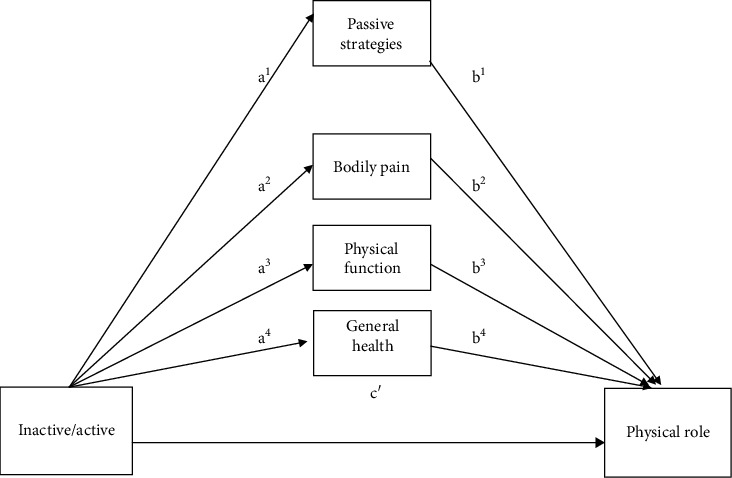
Hypothetical model of the relationship between inactive/active participants and physical role.

There are some factors such as physical function, bodily pain, passive strategies, and general health that could influence the relationship between PA and physical role; that is to say, these factors could act as mediator variables that explain the relationship between PA and physical role. Depending on the specific population group analyzed (inactive/active group), the influence of each variable could be different [35]. Therefore, in order to reach a deep understanding of the relationship between an active lifestyle and physical role in older women, the aims of this study were as follows: firstly, to analyze differences between inactive and active older women in terms of clinical and sociodemographic characteristics, active and passive strategies, and HRQoL; secondly, to study the associations between physical role, physical function, bodily pain, general health (SF-36 dimensions), and passive strategies; and thirdly, to determine if physical function, bodily pain, passive strategies, and general health are significant mediators in the link between being inactive or active and physical role (Figure 1) in a sample of rural older women from the Málaga province, southern Spain.

## 2. Materials and Methods

### 2.1. Study Design

In this study, a cross-sectional design was used, and the sample was selected using a nonproportional quota sampling.

### 2.2. Participants

The minimum sample size was calculated at a 95% confidence level based on the population of people with ages between 60 years old and older from three small villages of the province of Málaga. The population of those villages ranged between 2,000 and 5,000 inhabitants according to the information of the National Institute of Statistic from Spanish Government (http://www.ine.es/). Considering a statistical power of 80%, a type 1 error or alpha of 0.05, and sample population of older adult women over 60 years old (*n* = 1.011), the minimum recommended sample size needed was of 122 participants. The strategy to perform the sample size calculation followed Hamburg's formula [36].

**Figure 2:**
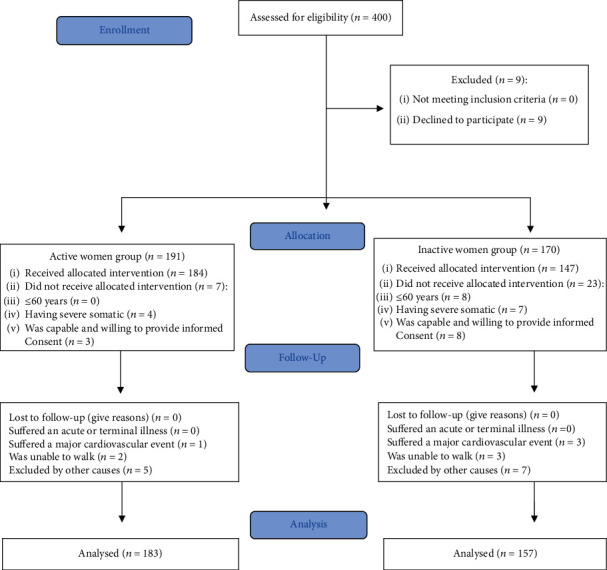
Participants' flow diagram (CONSORT 2010).

Two types of participants were included in this study: inactive and active older women (Figure 2). The inactive group did not practice any regular and controlled PA, and the active group included participants in a Pilates-aerobic exercise program of Málaga Provincial Government (Spain). The recruitment of the two groups was by telephone or by direct contact. It is important to highlight that the active group of participants was already attending a Pilates-aerobic exercise program when they were surveyed with the measurements cited before.

### 2.3. Procedure

Participants signed the informed consent form after they were provided with several details about the study such as the objective, duration, and procedure. After the participants agreed to take part in the study, it was corroborated if they were eligible to take part in the study. The inclusion criteria were the following: (i) not to be diagnosed with an acute or terminal illness, (ii) not to have functional mobility limitations. In order to avoid a low statistical power because of the sex division, male older participants were excluded because they represented only a small number. This study was conducted in accordance with the Declaration of Helsinki, and the protocol was approved by the University of Granada and Málaga Provincial Council, Sport Area.

The program's specific contents in the active older adult women group comprised upper and lower body strength exercises, agility, and especially the Pilates method (3-4 exercises of 8-12 repetitions regarding to the intensity of the session). Moreover, cardiorespiratory capacity and aerobic exercises were alternated trough 10 minutes with a moderate activity level. Cardiorespiratory capacity and aerobic exercises were always performed with music. The sessions were held twice a week in the afternoon and had an approximate duration of 45-60 minutes each, and the program lasted 24 weeks. Each PA was supervised by PA specialists. Physical activities were carried out in a covered room with temperature ranging between 17 and 22°C.

### 2.4. Measurements

#### 2.4.1. Health-Related Quality of Life

The Spanish version [7] of the Short-Form Health Survey-36 (SF-36) was used to assess HRQoL. This questionnaire comprises a total of 36 items that are grouped into eight dimensions: physical function, physical role, bodily pain, general health (physical component, all), vitality, social functioning, emotional role, and mental health (mental component, all). Each dimension score ranges from 0 to 100, where 0 indicates the worst possible health status and 100 the best possible. According to Angst et al. [37], the minimum clinically important difference (MCID) for the scores of all the dimensions of the SF-36 Health Survey should ranges between 30 and 50 points. Thus, the SF-36 dimensions with a score under 30 points should be rejected as study variable. In our study, there are three SF-36 dimensions with scores ranging between 30 and 50 points both in active participants and in inactive participants but always over 30 point score.

#### 2.4.2. Pain Coping Strategies

The Spanish version [19] of the Vanderbilt Pain Management Inventory (VPMI) [38] assessed the pain coping strategies, specifically passive and active strategies. These two scales comprise 18 items (active strategy = 7 items; passive strategy = 11 items) rated on a five-point scale that assesses how people cope with chronic pain.

Active coping entails that patients attempt to function despite their pain, while passive coping means that patients give the control of the pain to others or let the pain negatively affect other areas of their life.

The mean score of active and passive strategies (VPMI) obtained by the participants of this study is similar to the ones found in previous studies that have used the VPMI.

### 2.5. Statistical Analysis

Data were analyzed using the Statistical Package for Social Science program (IBM SPSS Statistics for Windows 21.0. Armonk, NY, USA). Significant differences between inactive and active participants in clinical characteristics, sociodemographic characteristics, active and passive pain coping strategies assessed with the VPMI, and the SF-36 dimensions were assessed by performing independent-sample Student's *t*-test. For the interpretation of the effect sizes of Student's *t*-tests, Cohen's *d* was calculated. The effect size can be interpreted as small (0.2 < *d* < 0.5), medium (0.5 < *d* < 0.8), or large (0.8 < *d*) [39].

In order to analyze the impact of being inactive/active on physical role, the SF-36, and the VPMI dimensions, a multiple stepwise regression analysis was performed. The linearity, independence (Durbin−Watson=bodily pain SF−36 dimension, between 0 and 4), normality, and lack of multicollinearity (the tolerance levels>.1 and the variance inflation factor<10) assumptions were met. The order of variables entered in the different steps was based on the study's aims and on findings from the literature on older people and PA that indicates the HRQoL level is a factor that allows health and symptomatic older people to cope with pain [40]. In this way, physical function was entered in the first step and subsequently bodily pain, general health (all, SF-36 dimensions), and passive strategies (VPMI dimension).

In order to assess whether the relationship between being inactive/active (independent variable) and physical role (dependent variable) was mediated by three dimensions of the SF-36 (bodily pain, physical function and general health) and passive strategies (see Figure 1), a multiple mediation analysis was performed using the bias-corrected bootstrapping approach [41]. Bootstrapping is a nonparametric resampling method used for estimating direct and indirect effects in multiple mediator models. This method involves repeatedly extracting samples from the data by randomly sampling with replacement and estimating the indirect effect in each resampled data-set [41]. The percentile bootstrap confidence intervals (CIs) are analyzed to establish whether mediation occurs. If zero is comprised within the 95% CIs, then the lack of significance is assumed [41, 42]. The bootstrapping method shows the advantage of analyzing multiple mediators simultaneously [41, 43] and minimizing the number of inferential tests which reduces the likelihood of Type I error [41]. Absence of collinearity assumptions (using correlation matrix, VIF, and eigenvalues) was also tested.

## 3. Results

The sample comprised 340 older women age 60 years old or older of which 157 were inactive participants (*M*_age_ = 69.9, SD = 7.1) while 183 were active participants and attended a Pilates-aerobic interventional program (*M*_age_ = 68.8, SD = 5.3). Statistically significant differences were found between inactive and active older adult women participant in VPMI strategies, passive strategies (inactive participants = 22.88 ± 6.73; active participants = 21.13 ± 6.07; *p* value = .019; *t* value = 2.350; and Cohen′s *d* = .27), and active strategies (inactive participants = 15.60 ± 4.79; active participants = 16.69 ± 4.53; *p* value = .041; *t* value = −2.050; and Cohen′s *d* = .23).

With regard to the differences in the SF-36 dimensions, statistically significant differences were also found between the two groups of participants of the study in physical function (inactive participants = 55.11 ± 25.82; active participants = 66.11 ± 25.13; *p* value = .001; *t* value = −3.781; and Cohen's *d* = .43), bodily pain (inactive participants = 45.46 + 29.18; active participants = 56.15 + 27.57; *p* value = .001; *t* value = −3.315; and Cohen′s *d* = .37), general health (inactive participants = 56.54 + 17.88; active participants = 46.07 + 19.37; *p* value = .001; *t* value = 4.923; and Cohen′s *d* = .56), vitality (inactive participants = 53.93 + 22.66; active participants = 44.48 + 21.15; *p* value = .001; *t* value = 3.781; and Cohen′s *d* = .43), mental health (inactive participants = 46.88 + 22.12; active participants = 36.48 + 20.53; *p* value = .001; *t* value = 4.264; and Cohen′s *d* = .48), and physical role (inactive participants = 44.12 + 44.79; active participants = 57.05 + 44.57; *p* value = .011; *t* value = −2.559; and Cohen′s *d* = .29).

In the multiple stepwise regression, bodily pain, physical function, and general health were significant predictors and explained 28.9%, 8.8%, and 1.6%, respectively, in the variance of physical role (see Table 1).

The multiple mediation analysis revealed that the paths from being active/inactive to physical function, bodily pain, general health, and passive strategies were significant. With regard to the paths from the proposed mediators to physical role, all four proposed mediators, mentioned previously, were significant (see Table 2). However, it was found that the direct effect of the path from inactive/active participants to physical role (*c*′) was not significant. Finally, the total effect of inactive/active participants on physical role (*c*) coefficient = 15.28; SE = 5.40, at 95%CI lower limit = 4.64, upper limit = 25.93; and *p* value = .01) was significant.

**Figure 3:**
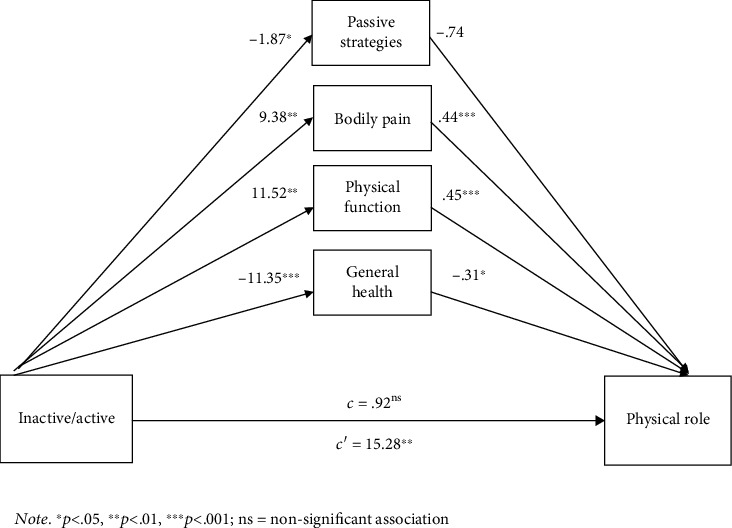
Total effect's results of being inactive/active participants on physical role by the mediators passive strategies, bodily pain, physical function and general health. Note: ∗*p* < .05, ∗∗*p* < .01, ∗∗∗*p* < .001; ns: nonsignificant association.

Therefore, an indirect effect was found from being active/inactive to physical role via the mediators passive strategies, bodily pain, physical function, and general health (ß = 14.36; at 95%CI lower limit = 7.48, upper limit = 21.14). The mediation estimated that 93.9% of the total effect of being inactive/active on physical role was mediated by passive strategies, bodily pain, physical function, and general health.  Figure 3 illustrates all paths and the possible significant results from being active/inactive to physical via the four mediators.

## 4. Discussion

The aims of this study were firstly to analyze differences between inactive and active older women in terms of clinical and sociodemographic characteristics, active and passive strategies, and HRQoL; secondly, to study the associations between physical role, physical function, bodily pain, general health (SF-36 dimensions), and passive strategies; and thirdly, to determine if physical function, bodily pain, passive strategies, and general health are significant mediators in the link between being inactive or active and physical role in a sample of rural older.

The relationship between physical exercise, physical function, and physical role is complex and results from the previous studies are contradictory. In addition, previous studies showed that the relationship and influence between these three variables may vary among women from different geographical locations [6, 44].

Results showed that both, inactive and active women, were similar in terms of age at which menopause occurred, weight, and educational level. Nevertheless, they differ in terms of the coping strategies they used to cope with pain. Active women used active pain coping strategies more frequently and presented a higher physical function, physical role, and bodily pain, but they obtained lower general health, vitality, and mental health scores than inactive women. These results are in agreement with previous studies on pain coping strategies used by older adults. In general, active older adults tend to actively cope with problems by engaging with the stressful event while sedentary older people, who tend to have a lower level of social participation, tend to use withdrawal and avoidance strategies more often [44].

Similarly, it seems logical to expect active older adults to have a better HRQoL [16, 45], to score higher in physical function [13–15] and in physical role [8], and to have lower levels of bodily pain [46] than inactive older people. However, contrary to our expectation, active older women in this sample had a more negative perception of their health status and lower scores on vitality and mental health measures than sedentary older women. These results are in disagreement with various previous studies that had found an association between PA and better subjective health status [47, 48], as well as higher scores on vitality and mental health measures [49]. However, active older women from the present study were selected from a PA program; thus, it might be possible that their main motivation to take part in the PA program was their negative perceptions of their general health status and a low level of vitality or a higher number of mental health problems. Because these variables were not measured before enrolling in the PA program, it was not possible for us to ascertain whether the perception regarding their health status and the vitality and mental health scores changed after taking part in the activity program. Thus, these scores might not be a consequence of taking part in a PA program but rather a previous characteristic of the women who enrolled in this program. Because this is a cross-sectional study, it limits the ability to establish causality in the relationships between the variables of interest. Thus, future studies should use longitudinal designs to test if PA has a positive or a negative effect on the aforementioned variables.

On the other hand, it was found that bodily pain, physical function, and general health were significant predictors of physical role, especially bodily pain explained 28.6% in the variance of physical role and physical function explained an additional 10.1%.

Results of previous studies on the relationship about bodily pain and physical role are contradictory. While Karsdorp et al. [21] had not found a relationship between bodily pain and the capability to day-to-day activities, a survey of chronic pain in Europe [50] indicated that pain is directly related to a decline in physical role in daily life activities. In the present work, it was found that higher bodily pain was related to a less ability to perform daily life activities, supporting results of the surveys' authors [50].

In addition, physical function and physical role are two variables that are related to a great extent. In order to be independent for performing daily living activities and work-related activities, a good physical function is essential [10, 51].

Finally, the results of the present study indicate that a higher general health is related to a lower score in the physical role dimension. This finding is contradictory to the previous scientific literature that conveys that a good general health is associated with a higher physical role of older people [6, 33]. These results could be explained by the fact that those older women who have a good general health status might find themselves unable to perform their daily activities when they have some minimum physical health problem; meanwhile, older people who live daily with a more weakened general health are more experienced in dealing with day-to-day activities despite these problems because they become more resilient, so their physical role score is higher. However, this affirmation is just speculative, and it needs to be tested in future studies.

With regard to the third objective of this study, the multiple mediation analysis showed that three of four proposed mediators (physical function, bodily pain, and general health) were significant in the link between being active/inactive and physical role whereas the fourth mediator (passive strategies) was only significant in the indirect effect of being inactive/active on passive strategies (effect of X on M). Limitation in performing daily living is influenced by the level of activity; thus, sedentary people have more functional limitations and therefore a lower score in physical role, while active people have fewer functional limitations. In addition, the relationship between being active/inactive and physical role could also be mediated by other variables such as the intensity of pain when performing physical activities and by physical function. In this way, active people might experience lower levels of pain and a better physical function, and this in turn could result in a better physical role. Previous researches postulated that physical exercise could influence how older people experience bodily pain [46] and also contributes to a better physical function [13, 14]. In turn, experiencing chronic pain when performing daily activities negatively influences physical role [28] and is also related to a lower physical function [10]; therefore, bodily pain and physical function were important mediators of the relationship between being active and physical role in older people.

Nevertheless, the path from being active/inactive to physical role via general health and passive strategies used to cope with pain showed an inverse relationship. Active older women in this sample used passive coping strategies to a lesser extent when coping with pain and had a lower general health. Results regarding the use of coping strategies are congruent with those found in previous studies. On the one hand, physically active older people usually use active or problem-focused coping strategies [44] and also use more active strategies to cope with pain [26]. On the other hand, the use of passive and avoidant pain coping strategies negatively influences the physical role [30] that is related with a negative interpretation of pain situation [19, 20], which could lead to a more negative interpretation of their own limitations to day-to-day activities, or their physical role.

Finally, contrary to what it was expected, general health was a significant mediator, in the association between active/inactive and physical role. Physical exercise was related to worse general health, and this, in turn, was linked with a better physical role. It is possible that the health problems were the ones that really led people to practice physical exercise as a way of pain coping with them, but since we do not have the results of the intervention program on this variable, we cannot verify this hypothesis.

## 5. Limitations

There are some limitations to the present study which affect the generalizability of the findings. Results of the regression analysis and of the multiple mediation analysis should be interpreted cautiously due to the cross-sectional design. We assumed PA, operationalized as being active or inactive, to predict the physical role, bodily pain, and general health to cope with pain as mediators in this relationship. However, it is also possible that physical role could affect an individual's pain coping strategies and his or her PA level. Therefore, future studies should test reciprocal models using longitudinal designs to test the link between PA and physical role. On the other hand, the use of nonprobability sampling and the fact that the sample was collected from rural settings and only women took part might not allow extrapolating the results of this study to the general Spanish population of older adults. However, the sample of this study was large, and it was selected at 95% level; thus, we can expect results from this study to be applicable at least to community-dwelling older women from rural areas in southern Spain. Hence, future research should explore the study variables in larger and more diverse samples of older people. Also, participants in the active group took part in Pilates and aerobic exercise; thus, other physical exercise activities such as tai-chi, progressive resistance training, or PA in the aquatic environment might have different effects on physical role [52]. Finally, questionnaires were used to assess participants' coping strategies and HRQoL. The use of self-reported measures could be biased by participants' goals and motives and shared method variance could determine inflation of the relationship between variables. In order to avoid possible bias attributed to self-report, future studies should contemplate supplementing self-reported measures with implicit methods and experience sampling.

## 6. Conclusions

In conclusion, this study contributes to a better understanding of the mechanisms that link physical exercise and physical role. Moreover, bodily pain, physical function, general health, and strategies used to cope with pain explain the effect that PA has on physical role.

This information is useful for developing interventions aimed at modifying older people's perception of the extent to which their health problems affect their performance of daily life and work-related activities. This is essential since a negative perception that individuals have of their ability to perform daily life and work-related activities could affect their HRQoL. Also, the participants' coping style in this study could predict a possible engagement in regular PA or not. Thus, it is necessary to avoid pain coping passive strategies among older women and motivate them through active strategies such as motivational interviewing, or a personalized tracking at home. This is especially important for sedentary older people who tend to use passive pain coping strategies when they have to face pain. This would contribute to their continuity in taking part in PA programs during longer periods of time and in even to modify their physical role.

## Figures and Tables

**Table 1:** Results of the multiple stepwise regression analysis for physical role with inactive/active, physical function, bodily pain, general health (SF-36), and passive strategies of VPMI.

	Step	Variable	*B*	*β*	*R*	*R* ^2^	Corrected *R*^2^	Change in *R*^2^
Physical role	1	Bodily pain	.846	.538∗∗∗	.538	.289	.287	.289∗∗∗
	2	Physical function	.561	.330∗∗∗	.614	.377	.372	.088∗∗∗
	3	General health	-.377	-.162∗	.627	.393	.386	.016∗∗

*Note:N* = 340; *B*, unstandardized coefficient; *β*, standardized coefficient; regression coefficients were significant at ∗*p* < .05, ∗∗*p* < .01, and ∗∗∗*p* < .001.

**Table 2:** Indirect effect of inactive/active on physical role through physical function, general health, bodily pain, and passive strategies.

Mediator	Effect of X on M (a^1^-a^4^)	SE	Effect of M on Y (b^1^-b^4^)	SE	Bootstrap estimate	Effect (*ß*)	BCa 95% CI	
							Lower	Upper
Passive strategies	-1.874∗(a^1^)	.769	-.743(b^1^)	.415	1.045	1.393	-.034	4.259
Bodily pain	9.379∗∗(a^2^)	3.444	.448∗∗∗(b^2^)	.097	1.928	4.202	1.202	8.774
Physical function	11.522∗∗∗(a^3^)	3.149	.455∗∗∗(b^3^)	.097	1.894	5.247	2.202	9.798
General health	-11.354∗∗∗(a^4^)	2.265	-.310∗(b^4^)	.145	1.886	3.519	.424	7.823

*Note*: ∗*p* < .05, ∗∗*p* < .01, and ∗∗∗*p* < .001.

## Data Availability

https://fairsharing.org/biodbcore-001683/
